# Changes in high-density lipoprotein cholesterol with risk of Cardiovascular Disease among initially high-density lipoprotein-high participants

**DOI:** 10.1186/s12933-023-01805-8

**Published:** 2023-03-28

**Authors:** Hye Jun Kim, Seogsong Jeong, Yun Hwan Oh, Sun Jae Park, Yoosun Cho, Sang Min Park

**Affiliations:** 1 Department of Biomedical Sciences, Seoul National University College of Medicine, Seoul, South Korea; 2 Department of Biomedical Informatics, CHA University School of Medicine, Seongnam, South Korea; 3 Department of Family Medicine, Chung-Ang University Gwangmyeong Hospital, Chung-Ang University College of Medicine, Gwangmyeong, Korea; 4 Total Healthcare Center, Kangbuk Samsung Hospital, Sungkyunkwan University School of Medicine, Seoul, Korea; 5 Department of Family Medicine, Seoul National University Hospital, Seoul, South Korea

**Keywords:** Cholesterol, HDL, Public health, Mass screening, Cardiovascular diseases, Coronary heart disease, Stroke.

## Abstract

**Background:**

High-density lipoprotein cholesterol’s (HDL-C) long-held status as a cardiovascular disease (CVD) preventative has been called into question. Most of the evidence, however, focused on either the risk of death from CVD, or on single time point level of HDL-C. This study aimed to determine the association between changes in HDL-C levels and incident CVD in individuals with high baseline HDL-C levels (≥ 60 mg/dL).

**Methods:**

77,134 people from the Korea National Health Insurance Service-Health Screening Cohort were followed for 517,515 person-years. Cox proportional hazards regression was used to evaluate the association between change in HDL-C levels and the risk of incident CVD. All participants were followed up until 31 December 2019, CVD, or death.

**Results:**

Participants with the greatest increase in their HDL-C levels had higher risks of CVD (adjusted hazard ratio [aHR], 1.15; 95% confidence interval [CI], 1.05–1.25) and CHD (aHR 1.27, CI 1.11–1.46) after adjusting for age, sex, household income, body mass index, hypertension, diabetes mellitus, dyslipidemia, smoking, alcohol consumption, moderate-to-vigorous physical activity, Charlson comorbidity index, and total cholesterol than those with the lowest increase in HDL-C levels. Such association remained significant even among participants with decreased low-density lipoprotein cholesterol (LDL-C) levels for CHD (aHR 1.26, CI 1.03–1.53).

**Conclusions:**

In people with already high HDL-C levels, additional increases in HDL-C levels may be associated with an increased risk of CVD. This finding held true irrespective of the change in their LDL-C levels. Increasing HDL-C levels may lead to unintentionally elevated risk of CVD.

**Supplementary Information:**

The online version contains supplementary material available at 10.1186/s12933-023-01805-8.

## Introduction

High-density lipoprotein cholesterol (HDL-C) levels were previously considered to be inversely associated with a higher risk of cardiovascular disease (CVD). [1] The risk of CVD drops by 2–3% for every unit rise in HDL-C levels. [2] However, HDL-C therapy, such as cholesteryl ester transfer protein inhibition, which raises its level, has been questioned in terms of CVD risk reduction. [3, 4] A recent study found that very high HDL-C levels are associated with unfavorable outcomes in patients with coronary heart disease (CHD), suggesting that HDL-C levels may worsen all-cause and cardiovascular mortality. [5] Very high levels of HDL-C have also been associated with a higher mortality rate, according to epidemiological studies of CVD-free populations in Canada and northern Europe. [6, 7]

The reverse cholesterol transport, which is predominantly driven by cholesterol efflux, is one of the protective effects of HDL against CVD. [8] Reverse cholesterol transport refers to the efflux of extracellular cholesterol from peripheral tissues back to the liver, where it is metabolized and then excreted in the bile and feces. [9] As a result of insufficient reverse transfer of cholesterol, which leaves extra cellular cholesterol in the vasculature, low HDL-C levels may increase the risk of atherosclerosis.

However, HDL-C levels may not always correspond to its functionality. [10] For example, a prospective study involving 8,267 general population found a lower CVD risk with an increased cholesterol efflux capacity (CEC) mediated by HDL-C, independently of their HDL-C levels. [11] Another finding has suggested that the CEC of HDL-C was substantially and inversely related to the prevalence of carotid and coronary atherosclerosis and with incident CVD, irrespective of HDL-C levels. [8] Additionally, unique phenotypes where CHD developed in the absence of traditional risk factors and with a very high HDL-C level have been observed, defying the commonly held belief that HDL-C plays a cardioprotective role. [10] Individuals with very high HDL-C levels may be more vulnerable to CVD, and lower CEC is thought to be one of the reasons. [10] Despite a number of studies challenging the role of HDL-C as a preventive factor against CVD, these studies have largely focused on a single time point level of HDL-C, typically the extremely high level above 80 mg/dL. Thus, more research is required to ascertain the effect of HDL-C level changes on CVD risk, especially among those with high but not very high levels of HDL-C. Hence, this study aimed to provide evidence as to whether an increase in HDL-C is related to incident CVD events among participants who already had a high HDL-C level. According to the 4th edition Korean guidelines for the management of dyslipidemia, HDL-C levels of 60 mg/dL or higher are considered high HDL-C levels and a protective factor against CVD. [12] Thus, this study focused on individuals with baseline HDL-C levels of ≥ 60 mg/dL.

## Methods

### Study population

This retrospective nationally representative cohort study obtained data from the Korea National Health Insurance Service (NHIS)-Health Screening Cohort (HEALS), which is a 10% random sampling of the entire NHIS database. The NHIS provides compulsory insurance services regarding all aspects of medical healthcare for Korean citizens. [13] The NHIS has been collecting information regarding sociodemographic characteristics, drug prescription records, treatment records, and health screening examination results, including questionnaires on lifestyle behaviors, serological characteristics, and anthropometric measurements. [13].

**Fig. 1:**
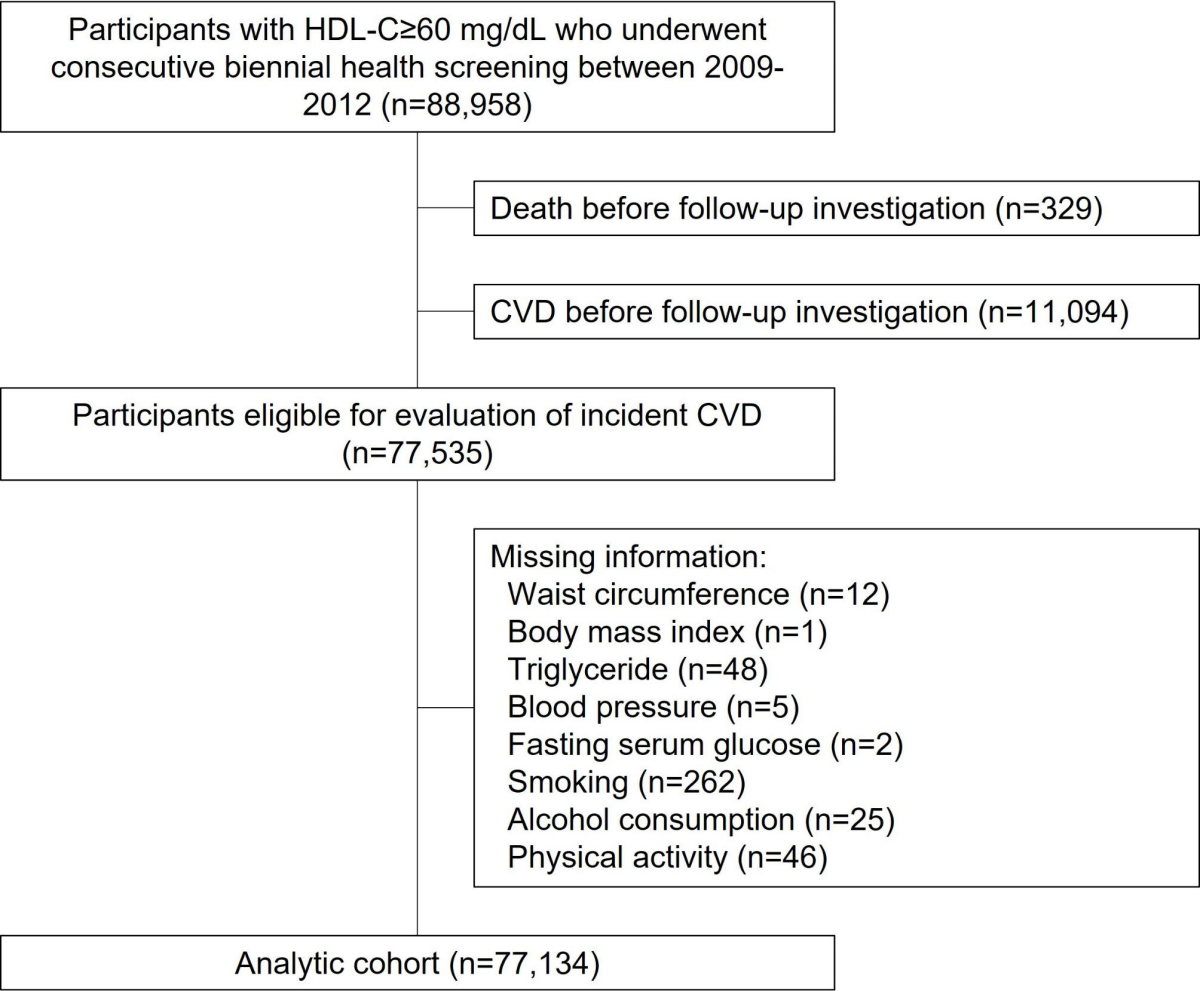
**Flow diagram for the inclusion of study participants.** The study population was derived after excluding participants without information for high-density lipoprotein cholesterol (HDL-C) level. Only participants who underwent at least two consecutive biennial health screening were enrolled to calculate the change in HDL-C levels

In the present study, we collected data on 88,958 participants with HDL-C ≥ 60 mg/dL aged ≥ 40 years who underwent at least one biennial health screening between 2011 and 2012 and had information of HDL-C from a health screening between 2009 and 2010. We first excluded those who died (n = 329) and had a history of CVD (n = 11,094) before the follow-up investigation. Those with missing information for the covariates were excluded (n = 401). Finally, the analytic cohort consisted of 77,134 participants aged at least 40 with complete information and without a history of CVD (Fig. 1). This study was conducted in accordance with STROBE guidelines. [14] The Institutional Review Board of the Seoul National University Hospital approved this study (No.: E-2108-136-1246). Due to the database’s anonymization per stringent confidentiality guidelines, the need for informed consents was waived.

### Follow-up for CVD

From January 1, 2013, all participants were tracked until either CVD, death, or 31 December, 2019. In accordance with the guidelines of the American Heart Association, [15] CVD was considered present when a participant had two or more days of hospitalization due to CHD (International Classification of Diseases Tenth Revision [ICD-10] codes, I20-I25) or stroke (ICD-10 codes, I60-I69). The operational definition of CVD in the NHIS is considered accurate and used in a number of previous studies. [16].

### Key variables

We considered the following variables as potential confounding factors for the adjusted analyses: age (continuous; years), sex (categorical; men and women), insurance premium as a proxy for household income (categorical; upper half and lower halves), body mass index (BMI; continuous; kg/m^2^), total cholesterol (continuous; mg/dL), hypertension (categorical; yes and no), type 2 diabetes mellitus (DM; categorical; yes and no), dyslipidemia (categorical; yes and no), smoking (categorical; never, past, and current), alcohol consumption (categorical; yes and no), moderate-to-vigorous physical activity (MVPA; categorical; 0, 1–2, 3–4, and ≥ 5 times/week), and Charlson comorbidity index (CCI; categorical; 0, 1–2, and ≥ 3).

The presence of the ICD-10 codes and prescription records for antihypertensive, antidiabetic, and antidyslipidemic drugs indicated the presence of hypertension (ICD-10 codes, I10), DM (ICD-10 codes, E10-E14), and dyslipidemia (ICD-10 codes, E78), respectively. The CCI was calculated as described in a previous study. [17].

### Statistical analysis

After calculating one crude model and three adjustment models, the Cox proportional hazards regression model was used to assess the relationship between HDL-C level changes and the risk of incident CVD. After accounting for age, the adjusted hazard ratio (aHR) and 95% confidence interval (CI) were calculated in the minimally adjusted model. Age, household income, BMI, hypertension, DM, dyslipidemia, smoking, alcohol consumption, MVPA, and CCI were all factors in the second adjustment model. The final adjustment model was further adjusted for total cholesterol. We also conducted competing risk analyses that set CHD and stroke as competing risks for each other using the Fine-Gray model.

Changes in HDL-C levels were calculated by subtracting HDL-C level in the health screening between 2011 and 2012 by HDL-C level in the health screening between 2009 and 2010. Both HDL-C levels at baseline and changes in HDL-C levels were stratified into quartiles using the ranking procedure. The incidence of CVD was calculated as the number of events per 1,000 people-years (PY). We conducted sensitivity analyses to control the on-developing CVD cases at the first time of follow-up investigation by excluding participants with CVD cases that occurred within 1, 2, and 3 years since the first date of follow-up, respectively. To assess the risk of CVD according to changes in HDL-C over a more extended period of time, we have collected HDL-C levels of the study population in health screenings between 2013 and 2014, and calculated the change in HDL-C levels between 2009 and 2010 and 2013–2014.

Stratified analyses were carried out for two purposes. The first purpose was to assess potential interactions between changes in HDL-C levels and key variables, whereas the second purpose was to identify a specific population whose CVD-free survival may not be affected by the elevation of HDL-C levels. We used age, sex, obesity, abdominal obesity, smoking, alcohol consumption, MVPA, and CCI in the stratification of the analytic cohort. The significance level was considered as a two-tailed *P* value of less than 0.05. SAS version 9.4 (SAS Institute Inc., Cary, NC, USA) and R software version 3.3.3 (R Development Core Team, Vienna, Austria) were used to perform all data mining and statistical analyses.

## Results

A total of 4,194 CVD events (5.4%) were identified during the follow-up period of up to 517,515 person-years. Table 1 shows the descriptive characteristics of the study participants. Out of a total of 77,134 participants with a median (interquartile range [IQR]) age of 56 (52–63) years, 43,720 (56.7%) were women. Men tended to have higher proportions of upper half household income, hypertension, DM, smoking, alcohol consumption, and MVPA. In addition, men had higher levels of waist circumference, systolic and diastolic blood pressure, triglyceride, alanine aminotransferase, aspartate aminotransferase, and γ-glutamyl transpeptidase.

**Table 1:** Descriptive characteristics of the participants

Characteristic	Participant (n = 77,134)	Men (n = 33,414)	Women (n = 43,720)	*P* value
Age, years	56 (52–63)	56 (52–63)	56 (52–62)	< 0.001
Household income^a^, n (%)				< 0.001
Upper half	47,747 (61.9)	22,935 (68.6)	24,812 (56.8)	
Lower half	29,387 (38.1)	10,479 (31.4)	18,908 (43.2)	
Body mass index, kg/m^2^	23.0 (21.2–25.0)	23.0 (21.2–24.9)	23.0 (21.2–25.0)	< 0.001
Waist circumference, cm	79 (73–84)	82 (77–87)	76 (71–82)	< 0.001
Systolic blood pressure, mmHg	122 (113–133)	126 (117–135)	120 (110–130)	< 0.001
Diastolic blood pressure, mmHg	78 (70–82)	80 (70–85)	75 (70–80)	< 0.001
Triglyceride, mg/dL	90 (66–124)	94 (68–133)	87 (65–117)	< 0.001
Total cholesterol, mg/dL	207 (185–232)	201 (180–226)	211 (189–236)	< 0.001
HDL cholesterol, mg/dL	67 (63–74)	67 (63–74)	68 (63–75)	< 0.001
Alanine aminotransferase, IU/L	19 (15–26)	21 (16–29)	18 (14–24)	< 0.001
Aspartate aminotransferase, IU/L	24 (20–29)	25 (21–31)	23 (20–27)	< 0.001
γ-glutamyl transpeptidase, IU/L	22 (15–36)	32 (21–57)	17 (13–24)	< 0.001
Hypertension, n (%)	22,386 (29.0)	10,055 (30.1)	12,331 (28.2)	< 0.001
Diabetes mellitus, n (%)	5,544 (7.2)	2,946 (8.8)	2,598 (5.9)	< 0.001
Dyslipidemia, n (%)	17,064 (22.1)	5,536 (16.6)	11,528 (26.4)	< 0.001
Cigarette smoking, n (%)				< 0.001
Never smoker	54,744 (71.0)	11,867 (35.5)	42,877 (98.1)	
Former smoker	12,948 (16.8)	12,631 (37.8)	317 (0.7)	
Current smoker	9,442 (12.2)	8,916 (26.7)	526 (1.2)	
Alcohol consumption, n (%)				< 0.001
Yes	31,593 (41.0)	23,852 (71.4)	7,741 (17.7)	
No	45,541 (59.0)	9,562 (28.6)	35,979 (82.3)	
MVPA, n (%)				< 0.001
0 time/week	34,544 (44.8)	12,345 (36.9)	22,199 (50.8)	
1–2 time/week	12,482 (16.2)	5,977 (17.9)	6,505 (14.9)	
3–4 time/week	11,174 (14.5)	5,524 (16.5)	5,650 (12.9)	
≥ 5 time/week	18,934 (24.5)	9,568 (28.6)	9,366 (21.4)	
Charlson comorbidity index, n (%)				< 0.001
0	28,804 (37.3)	13,799 (41.3)	15,005 (34.3)	
1	25,965 (33.7)	10,799 (32.3)	15,166 (34.7)	
≥ 2	22,365 (29.0)	8,816 (26.4)	13,549 (31.0)	

Data are presented as median (interquartile range) unless otherwise specified

^a^Proxy for socioeconomic status based on the insurance premium of the National Health Insurance Service

Acronyms: HDL, high-density lipoprotein; MVPA, moderate-to-vigorous physical activity

The associations of HDL-C levels with the risk of incident CVD in overall participants, men, and women are presented in Supplemental Tables 1, Supplemental Tables 2, and Supplemental Table 3. We found no association of HDL-C levels with CVD risk at a single time point. Changes in HDL-C levels, on the other hand, were significantly associated with an increased risk of CVD (Table 2; *P* for trend = 0.015). The fourth quartile of the change in HDL-C levels showed a higher risk of CVD compared to the first quartile (aHR, 1.15; 95% CI, 1.05–1.25). The associations of changes in HDL-C levels with CVD with respect to men and women presented similar trends and results (Supplemental Tables 4 and Supplemental Table 5). In the competing risk analysis of CHD and stroke, a significant trend of a higher risk was found for both CHD and stroke by an increase of change in HDL-C, and the elevated risk was more obvious for stroke as compared to CHD.

**Table 2:** Association of change in high-density lipoprotein cholesterol levels with incident cardiovascular disease

	1st quartile (n = 19,457)	2nd quartile (n = 19,802)	3rd quartile (n = 18,890)	4th quartile (n = 18,966)	*P* for trend
Range, mg/dL	≤-2	-1 to + 6	+ 7 to + 14	≥+15	
Cardiovascular disease					
PY	130,976	133,502	126,844	126,065	
Event (%)	945 (4.9)	1,012 (5.1)	1,030 (5.5)	1,206 (6.4)	
Incidence/1,000 PY	7.2	7.6	8.1	9.6	
HR (95% CI)	1.00 (reference)	1.05 (0.96–1.15)	1.13 (1.03–1.23)	1.33 (1.22–1.45)	< 0.001
aHR (95% CI)^a^	1.00 (reference)	1.05 (0.96–1.14)	1.10 (1.01–1.20)	1.20 (1.10–1.31)	< 0.001
aHR (95% CI)^b^	1.00 (reference)	1.05 (0.96–1.15)	1.09 (1.00–1.19)	1.16 (1.07–1.26)	0.006
aHR (95% CI)^c^	1.00 (reference)	1.05 (0.96–1.15)	1.08 (0.99–1.18)	1.15 (1.05–1.25)	0.015
Coronary heart disease					
PY	132,730	135,197	128,632	128,092	
Event (%)	341 (1.8)	413 (2.1)	395 (2.1)	491 (2.6)	
Incidence/1,000 PY	2.6	3.1	3.1	3.8	
HR (95% CI)	1.00 (reference)	1.19 (1.03–1.37)	1.20 (1.03–1.38)	1.49 (1.30–1.71)	< 0.001
aHR (95% CI)^a^	1.00 (reference)	1.18 (1.02–1.36)	1.17 (1.01–1.35)	1.36 (1.19–1.57)	< 0.001
aHR (95% CI)^b^	1.00 (reference)	1.19 (1.03–1.37)	1.15 (0.99–1.32)	1.30 (1.13–1.49)	0.004
aHR (95% CI)^c^	1.00 (reference)	1.18 (1.02–1.36)	1.14 (0.98–1.32)	1.27 (1.11–1.46)	0.007
aHR (95% CI)^d^	1.00 (reference)	0.93 (0.83–1.04)	1.06 (0.95–1.18)	1.07 (0.96–1.19)	0.040
Stroke					
PY	131,981	134,725	128,039	127,417	
Event (%)	630 (3.2)	642 (3.2)	673 (3.6)	759 (4.0)	
Incidence/1,000 PY	4.8	4.8	5.3	6.0	
HR (95% CI)	1.00 (reference)	1.00 (0.89–1.11)	1.10 (0.99–1.23)	1.25 (1.12–1.39)	< 0.001
aHR (95% CI)^a^	1.00 (reference)	1.00 (0.89–1.11)	1.08 (0.97–1.20)	1.12 (1.01–1.25)	0.077
aHR (95% CI)^b^	1.00 (reference)	1.01 (0.90–1.12)	1.07 (0.96–1.20)	1.09 (0.98–1.22)	0.243
aHR (95% CI)^c^	1.00 (reference)	1.01 (0.90–1.12)	1.07 (0.96–1.19)	1.08 (0.98–1.21)	0.318
aHR (95% CI)^d^	1.00 (reference)	1.17 (1.01–1.35)	1.11 (0.96–1.29)	1.26 (1.09–1.46)	0.013

HR calculated using the Cox proportional hazards model

^a^Adjusted for age and sex

^b^Adjusted for age, sex, household income, body mass index, hypertension, diabetes mellitus, dyslipidemia, smoking, alcohol consumption, moderate-to-vigorous physical activity, and Charlson comorbidity index

^c^Adjusted for age, sex, household income, body mass index, hypertension, diabetes mellitus, dyslipidemia, smoking, alcohol consumption, moderate-to-vigorous physical activity, Charlson comorbidity index, and total cholesterol

^d^Assessed using the competing risk model to calculate the subdistribution hazard ratio after adjustments for variables in the model C with coronary heart disease or stroke as competing risks for each other

Acronyms: PY, person-year; HR, hazard ratio; CI, confidence interval; aHR, adjusted hazard ratio

When evaluated the associations of changes in HDL-C levels in a longer period of time by subtracting HDL-C levels between 2009 and 2010 from HDL-C levels between 2013 and 2014, the 4th quartile of the change in HDL-C levels revealed a higher risk of CVD compared to the 1st quartile (Supplementary Table 6; aHR, 1.24; 95% CI, 1.03–1.50).

The sensitivity analyses that calculated CVD risk after omitting incidents that occurred within 1, 2, and 3 years of the start of the follow-up are shown in Supplemental Table 7. The results supported the primary finding that the risks of CVD were significantly higher in fourth quartile groups compared to the corresponding first quartile groups in all three different washout periods.

**Fig. 2:**
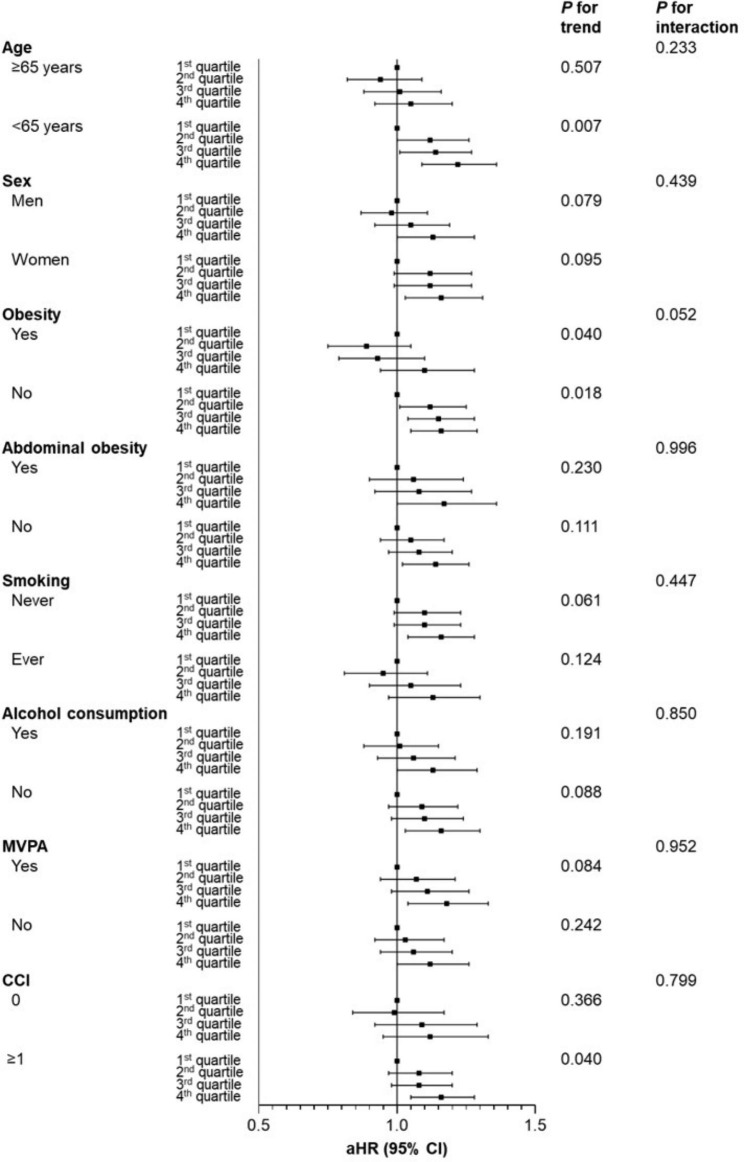
**Stratified analyses on association of the changes in high-density lipoprotein cholesterol levels with cardiovascular disease.** Adjusted hazard ratio calculated using Cox proportional hazards regression after adjustments for age, sex, household income, body mass index, total cholesterol, hypertension, diabetes mellitus, dyslipidemia, smoking, alcohol consumption, moderate-to-vigorous physical activity, and Charlson comorbidity index

The results of subgroup analyses are given in Fig. 2. A significant upward trend in the risk of CVD was found for participants with < 65 years of age and CCI ≥ 1. Among non-obese participants, the risk of CVD was significantly higher for those in the 2nd and 3rd quartiles for changes in HDL-C levels compared to the 1st quartile. In contrast, there was no difference in CVD risk between the 2nd and 3rd quartiles of obese participants compared to the 1st quartile.

Table 3 shows the associations of changes in HDL-C levels with risk of CVD according to the changes in low-density lipoprotein cholesterol (LDL-C) levels. Among participants with decreased LDL-C levels, greater changes in HDL-C levels showed a higher CVD risk. Participants in the 4th quartile of change in HDL-C compared to the 1st quartile had a higher risk of CHD (aHR, 1.34; 95% CI, 1.10–1.63; *P* = 0.003) and stroke (aHR, 1.18; 95% CI, 1.01–1.37; *P* = 0.034). The risk of CVD was then evaluated using the changes in HDL-C and LDL-C levels between 2009 and 2010 and 2013–2014. Among participants with increased LDL-C levels, the 4th quartile of change in HDL-C showed a higher risk of CVD compared to the 1st quartile (Supplementary Table 8; aHR, 1.39; 95% CI, 1.06–1.82; *P* = 0.016).

**Table 3:** Association of change in high-density lipoprotein cholesterol levels with incident cardiovascular disease according to the change in low-density lipoprotein cholesterol levels

	Change in HDL-C (mg/dL)				*P* for trend
	1st quartile, ≤-2	2nd quartile, -1 to + 6	3rd quartile, + 7 to + 14	4th quartile, ≥+15	
Cardiovascular disease					
LDL-C decrease^a^					
aHR (95% CI)	1.00 (reference)	1.04 (0.92–1.18)	1.05 (0.93–1.19)	1.10 (0.98–1.25)	0.441
*P* value		0.551	0.441	0.109	
LDL-C increase^b^					
aHR (95% CI)	1.00 (reference)	1.07 (0.94–1.21)	1.13 (1.00–1.27)	1.23 (1.09–1.39)	0.008
*P* value		0.319	0.060	< 0.001	
Coronary heart disease					
LDL-C decrease^a^					
aHR (95% CI)	1.00 (reference)	1.28 (1.04–1.57)	1.23 (1.00–1.51)	1.26 (1.03–1.53)	0.078
*P* value		0.018	0.054	0.024	
LDL-C increase^b^					
aHR (95% CI)	1.00 (reference)	1.09 (0.89–1.34)	1.07 (0.87–1.31)	1.34 (1.10–1.63)	0.017
*P* value		0.390	0.543	0.003	
Stroke					
LDL-C decrease^a^					
aHR (95% CI)	1.00 (reference)	0.95 (0.81–1.11)	0.97 (0.82–1.13)	1.03 (0.88–1.19)	0.735
*P* value		0.515	0.669	0.735	
LDL-C increase^b^					
aHR (95% CI)	1.00 (reference)	1.06 (0.91–1.24)	1.18 (1.02–1.37)	1.18 (1.01–1.37)	0.077
*P* value		0.430	0.028	0.034	

Data are hazard ratio (95% confidence interval) calculated using the Cox proportional hazards model after adjustments for age, sex, household income, body mass index, hypertension, diabetes mellitus, dyslipidemia, smoking, alcohol consumption, moderate-to-vigorous physical activity, and Charlson comorbidity index

^a^With change in LDL-C level of < 0 in the second health screening (2011–2012) compared to the first health screening (2009–2010)

^b^With change in LDL-C level of ≥ 0 in the second health screening (2011–2012) compared to the first health screening (2009–2010)

Acronyms: HDL-C, high-density lipoprotein cholesterol; LDL-C, low-density lipoprotein cholesterol

## Discussion

Results from this retrospective study of healthy individuals with HDL-C levels at baseline of 60 mg/dL or higher showed that those with the greatest increases in HDL-C had a 1.15-fold higher risk of developing CVD. This association was further validated by the change in HDL-C in a longer period of time of approximately 4 years. While there was a significant association between increased HDL-C levels and incident CHD, no such association could be shown for stroke. Remarkably, the risk of CHD was found to be elevated even in those whose LDL-C levels had been lowered. Additional increases in HDL-C may have a detrimental effect on CVD risk in individuals whose HDL-C levels are already quite high, regardless of their LDL-C levels. People under the age of 65, those who were not obese, and those who had at least one comorbid condition were also at higher risk of CVD when their HDL-C levels were increased. These findings lend support to previous studies that challenged the traditional HDL-C hypothesis, which proposes a causal linkage between HDL-C levels and the protection against CVD. [18].

A few studies have demonstrated an association between very high HDL-C levels (> 80 mg/dL) and unfavorable outcomes in the general population without known CHD. [6, 7] Recent studies have also linked very high HDL-C levels to a higher cardiovascular mortality rate in people with CHD. [5, 19] Nonetheless, the vast majority of these studies focused on HDL-C in a single level context, typically very high HDL-C levels above 80 mg/dL, and primarily CVD-related mortality risk. This meant that people whose HDL-C levels were already high but not extremely so, as well as those who experienced a change in their HDL-C levels, were largely ignored when considering the association between high HDL-C and CVD risk. To fill this knowledge gap, the current study analyzed the association between CVD incidence and changes in HDL-C levels, focusing on those with HDL-C levels of 60 mg/dL or higher. We found that having increased HDL-C levels is associated with a higher risk of CVD, and in particular CHD.

Premature CHD patients who had very high HDL-C levels had significantly lower CEC, according to a cross-sectional study. [10] Interactions with ATP-binding cassette (ABC) transporters, such as the macrophage cholesterol exporter ABCA1, are critical to the cholesterol effluxing functions of HDL-C. [18, 20] ApoA-I, the major protein component of HDL, is responsible for many of its beneficial effects on vascular health. [21] Researchers have found that apoA-I levels are predictive of future CVD risk and have an inverse relationship with the risk of major cardiovascular events. [22] Hence, apoA-I has been proposed as a more effective biomarker than HDL-C in determining CVD risk. [22] It can, however, be damaged by oxidative environment. [23] In particular, CEC of apoA-I is significantly reduced by chlorination via the myeloperoxidase pathway. [24] In a cell-based model, myeloperoxidase-induced chlorination hindered apoA-I from promoting cellular CEC by blocking its direct interaction with ABCA1 and activating the Janus kinase-2 signaling pathway. [20] Oxidative modification can also damage the binding site of lecithin–cholesterol acyltransferase (LCAT) in apoA-I. [25] Since LCAT is an essential enzyme in the cholesterol esterification and HDL-C maturation processes, any impairment in LCAT could worsen CEC of HDL-C. [26] Moreover, dysfunctional apoA-I due to oxidization contains oxindolyl alanine moiety at Trp72 (oxTrp72-apoA-I), which are pro-inflammatory properties. [21] It can reduce HDL biogenesis and cause inflammation. Higher oxTrp72-apoA-I levels in humans were associated with an increased risk of CVD. [21].

Besides apoA-I, several other HDL-C-associated proteins, such as complement factors, are involved in the acute-phase response. [23] Some of these include the iron-binding protein hemopexin and the haptoglobin that binds the hemoglobin that is released from the red blood cells. [27] The concentration of hemoglobin, haptoglobin, and hemopexin in HDL-C is strongly and positively correlated with lipid hydroperoxide levels. [28] CHD patients with these associations are more likely to experience systemic inflammation caused by the inflammatory properties of HDL-C. [28] Therefore, it is hypothesized that HDL-C can convert from an anti-inflammatory to a pro-inflammatory particle, [25] increasing the risk of CVD, [28] via a pathway involving the association of hemoglobin-hemopexin complexes with apoA-I. This may partially explain why participants with increased HDL-C levels had a higher risk of CHD regardless of the change in their LDL-C levels in this study. In addition, a previous study found that HDL-C could be pro-inflammatory in patients with CHD independent of the LDL-C concentration. [29].

To the best of our knowledge, this study is the first large-scale population-based longitudinal cohort study to define the association between changes in HDL-C levels and the risk of CVD among individuals with high baseline levels of HDL-C. We could expand on earlier studies that could not reach a consensus on the effect of high HDL-C on incident CVD. In addition, we filled a knowledge gap by focusing on individuals with high levels of HDL-C and their change of HDL-C levels, which had been overlooked in previous studies due to their focus on single time point levels of HDL-C, especially below normal (40 mg/dL) or very high (> 80 mg/dL) levels. Despite some notable strengths, the underlying limitations need to be considered before interpreting the results. Firstly, there may be an issue of reverse causality between HDL-C increase and risk of CVD due to the retrospective nature. Secondly, additional validations are needed before the results can be applied to people of a different race or ethnicity. Lastly, while this study focused on HDL-C levels, other important aspects of HDL-C, such as particle sizes or functions, could not be accounted. More research using in-depth biological information about HDL-C may help elucidating the biological mechanisms linking increased HDL-C levels and the onset of CVD.

## Conclusions

In this population-based longitudinal cohort study of participants with high HDL-C levels, the greatest HDL-C increase group revealed a higher risk of CVD by 1.15-fold. Notably, this association persisted even in those whose LDL-C levels were decreased. Participants showed a nearly 1.3-fold increased risk of incident CHD despite having decreased LDL-C levels. To enhance preventive management against CVD, redefining the effects of HDL-C level on CVD risk may be indispensable.

## Electronic supplementary material

Below is the link to the electronic supplementary material.

Supplemental Table 1: Association of high-density lipoprotein cholesterol quartiles with incident cardiovascular disease. Supplemental Table 2: Association of high-density lipoprotein cholesterol quartiles with incident cardiovascular disease among men. Supplemental Table 3: Association of high-density lipoprotein cholesterol quartiles with incident cardiovascular disease among women. Supplemental Table 4: Association of change in high-density lipoprotein cholesterol levels with incident cardiovascular disease among men. Supplemental Table 5: Association of change in high-density lipoprotein cholesterol levels with incident cardiovascular disease among women. Supplemental Table 6: Association of change in high-density lipoprotein cholesterol levels between 2009-2010 and 2013-2014 with incident cardiovascular disease. Supplemental Table 7: Sensitivity analyses on association of change in high-density lipoprotein cholesterol levels with incident cardiovascular disease. Supplemental Table 8: Association of change in high-density lipoprotein cholesterol levels between 2009-2010 and 2013-2014 with incident cardiovascular disease according to the change in low-density lipoprotein cholesterol levels between 2009-2010 and 2013-2014.

## Data Availability

The datasets generated and/or analyzed during the current study are available from the Korean National Health Insurance Service, https://nhiss.nhis.or.kr.

## Abbreviations

HDL-C: High-density lipoprotein cholesterol
CVD: Cardiovascular disease
CHD: Coronary heart disease
CEC: Cholesterol efflux capacity
NHIS-HEALS: Korea National Health Insurance Service-Health Screening Cohort
BMI: Body mass index
DM: Type 2 diabetes mellitus
aHR: Adjusted hazard ratio
CI: Confidence interval
LDL-C: Low-density lipoprotein cholesterol
