# Supplementary material for: Changes in high-density lipoprotein cholesterol with risk of Cardiovascular Disease among initially high-density lipoprotein-high participants
Source: Cardiovasc Diabetol. 2023 Mar 28;22:71. doi: 10.1186/s12933-023-01805-8 (PMC10053475; doi:10.1186/s12933-023-01805-8)
Supplement: Supplementary file 1 — Supplemental Table 1: Association of high-density lipoprotein cholesterol quartiles with incident cardiovascular disease. Supplemental Table 2: Association of high-density lipoprotein cholesterol quartiles with incident cardiovascular disease among men. Supplemental Table 3: Association of high-density lipoprotein cholesterol quartiles with incident cardiovascular disease among women. Supplemental Table 4: Association of change in high-density lipoprotein cholesterol levels with incident cardiovascular disease among men. Supplemental Table 5: Association of change in high-density lipoprotein cholesterol levels with incident cardiovascular disease among women. Supplemental Table 6: Association of change in high-density lipoprotein cholesterol levels between 2009-2010 and 2013-2014 with incident cardiovascular disease. Supplemental Table 7: Sensitivity analyses on association of change in high-density lipoprotein cholesterol levels with incident cardiovascular disease. Supplemental Table 8: Association of change in high-density lipoprotein cholesterol levels between 2009-2010 and 2013-2014 with incident cardiovascular disease according to the change in low-density lipoprotein cholesterol levels between 2009-2010 and 2013-2014. [file 12933_2023_1805_MOESM1_ESM.docx]

**SUPPLEMENTAL MATERIAL**

(page 2) Supplemental Table 1: Association of high-density lipoprotein cholesterol quartiles with incident cardiovascular disease

(page 3) Supplemental Table 2: Association of high-density lipoprotein cholesterol quartiles with incident cardiovascular disease among men

(page 4) Supplemental Table 3: Association of high-density lipoprotein cholesterol quartiles with incident cardiovascular disease among women

(page 5) Supplemental Table 4: Association of change in high-density lipoprotein cholesterol levels with incident cardiovascular disease among men

(page 6) Supplemental Table 5: Association of change in high-density lipoprotein cholesterol levels with incident cardiovascular disease among women

(page 7) Supplemental Table 6: Association of change in high-density lipoprotein cholesterol levels between 2009-2010 and 2013-2014 with incident cardiovascular disease

(page 8) Supplemental Table 7: Sensitivity analyses on association of change in high-density lipoprotein cholesterol levels with incident cardiovascular disease

(page 9) Supplemental Table 8: Association of change in high-density lipoprotein cholesterol levels between 2009-2010 and 2013-2014 with incident cardiovascular disease according to the change in low-density lipoprotein cholesterol levels between 2009-2010 and 2013-2014

**Supplemental Table 1.** Association of high-density lipoprotein cholesterol quartiles with incident cardiovascular disease

|  | **1^st^ quartile (n=17,315)** | **2^nd^ quartile (n=21,674)** | **3^rd^ quartile (n=18,996)** | **4^th^ quartile (n=19,149)** | ***P* for trend** |
| --- | --- | --- | --- | --- | --- |
| Range, mg/dL | ≤62 | 63-67 | 68-74 | ≥75 |  |
| Cardiovascular disease |  |  |  |  |  |
| PY | 116,160 | 145,568 | 127,266 | 128,521 |  |
| Event (%) | 966 (5.6) | 1,190 (5.5) | 1,057 (5.6) | 981 (5.1) |  |
| Incidence/1,000 PY | 8.3 | 8.2 | 8.3 | 7.6 |  |
| HR (95% CI) | 1.00 (reference) | 0.98 (0.90-1.07) | 1.00 (0.92-1.09) | 0.92 (0.84-1.00) | 0.181 |
| aHR (95% CI)^a^ | 1.00 (reference) | 1.00 (0.92-1.09) | 1.03 (0.95-1.13) | 0.96 (0.88-1.05) | 0.484 |
| aHR (95% CI)^b^ | 1.00 (reference) | 1.00 (0.92-1.09) | 1.05 (0.97-1.15) | 0.99 (0.91-1.09) | 0.504 |
| aHR (95% CI)^c^ | 1.00 (reference) | 1.00 (0.92-1.09) | 1.04 (0.95-1.14) | 0.97 (0.89-1.06) | 0.441 |
| Coronary heart disease |  |  |  |  |  |
| PY | 117,798 | 147,510 | 129,236 | 130,238 |  |
| Event (%) | 386 (2.2) | 501 (2.3) | 395 (2.1) | 358 (1.9) |  |
| Incidence/1,000 PY | 3.3 | 3.4 | 3.1 | 2.7 |  |
| HR (95% CI) | 1.00 (reference) | 1.04 (0.91-1.18) | 0.93 (0.81-1.07) | 0.84 (0.73-0.97) | 0.015 |
| aHR (95% CI)^a^ | 1.00 (reference) | 1.05 (0.92-1.20) | 0.97 (0.84-1.11) | 0.89 (0.77-1.02) | 0.087 |
| aHR (95% CI)^b^ | 1.00 (reference) | 1.06 (0.93-1.21) | 0.99 (0.86-1.14) | 0.93 (0.81-1.08) | 0.311 |
| aHR (95% CI)^c^ | 1.00 (reference) | 1.06 (0.92-1.21) | 0.98 (0.85-1.12) | 0.90 (0.78-1.04) | 0.146 |
| Stroke |  |  |  |  |  |
| PY | 117,301 | 147,046 | 128,416 | 129,526 |  |
| Event (%) | 613 (3.5) | 726 (3.3) | 705 (3.7) | 661 (3.5) |  |
| Incidence/1,000 PY | 5.2 | 4.9 | 5.5 | 5.1 |  |
| HR (95% CI) | 1.00 (reference) | 0.95 (0.85-1.05) | 1.05 (0.94-1.17) | 0.98 (0.88-1.09) | 0.235 |
| aHR (95% CI)^a^ | 1.00 (reference) | 0.96 (0.86-1.07) | 1.09 (0.97-1.21) | 1.02 (0.92-1.14) | 0.141 |
| aHR (95% CI)^b^ | 1.00 (reference) | 0.96 (0.87-1.07) | 1.10 (0.99-1.23) | 1.04 (0.93-1.16) | 0.070 |
| aHR (95% CI)^c^ | 1.00 (reference) | 0.96 (0.86-1.07) | 1.09 (0.98-1.22) | 1.02 (0.92-1.14) | 0.100 |

HR calculated using the Cox proportional hazards model.

^a^Adjusted for age.

^b^Adjusted for age, household income, body mass index, hypertension, diabetes mellitus, dyslipidemia, smoking, alcohol consumption, moderate-to-vigorous physical activity, and Charlson comorbidity index.

^c^Adjusted for age, household income, body mass index, hypertension, diabetes mellitus, dyslipidemia, smoking, alcohol consumption, moderate-to-vigorous physical activity, Charlson comorbidity index, and total cholesterol.

Acronyms: PY, person-year; HR, hazard ratio; CI, confidence interval; aHR, adjusted hazard ratio.

**Supplemental Table 2.** Association of high-density lipoprotein cholesterol quartiles with incident cardiovascular disease among men

|  | **1^st^ quartile (n=8,081)** | **2^nd^ quartile (n=8,124)** | **3^rd^ quartile (n=8,829)** | **4^th^ quartile (n=8,380)** | ***P* for trend** |
| --- | --- | --- | --- | --- | --- |
| Range, mg/dL | ≤62 | 63-66 | 67-73 | ≥74 |  |
| Cardiovascular disease |  |  |  |  |  |
| PY | 53,717 | 53,878 | 58,399 | 55,158 |  |
| Event (%) | 491 (6.1) | 538 (6.6) | 561 (6.4) | 527 (6.3) |  |
| Incidence/1,000 PY | 9.1 | 10.0 | 9.6 | 9.6 |  |
| HR (95% CI) | 1.00 (reference) | 1.09 (0.97-1.24) | 1.05 (0.93-1.19) | 1.05 (0.93-1.18) | 0.566 |
| aHR (95% CI)^a^ | 1.00 (reference) | 1.09 (0.96-1.23) | 1.03 (0.91-1.17) | 1.01 (0.89-1.14) | 0.532 |
| aHR (95% CI)^b^ | 1.00 (reference) | 1.09 (0.97-1.24) | 1.05 (0.93-1.18) | 1.04 (0.92-1.18) | 0.553 |
| aHR (95% CI)^c^ | 1.00 (reference) | 1.09 (0.96-1.23) | 1.03 (0.91-1.17) | 1.01 (0.89-1.14) | 0.511 |
| Coronary heart disease |  |  |  |  |  |
| PY | 54,420 | 54,660 | 59,337 | 56,013 |  |
| Event (%) | 220 (2.7) | 258 (3.2) | 229 (2.6) | 216 (2.6) |  |
| Incidence/1,000 PY | 4.0 | 4.7 | 3.9 | 3.9 |  |
| HR (95% CI) | 1.00 (reference) | 1.17 (0.98-1.40) | 0.96 (0.79-1.15) | 0.95 (0.79-1.15) | 0.080 |
| aHR (95% CI)^a^ | 1.00 (reference) | 1.16 (0.97-1.39) | 0.94 (0.78-1.13) | 0.93 (0.77-1.12) | 0.055 |
| aHR (95% CI)^b^ | 1.00 (reference) | 1.17 (0.98-1.40) | 0.96 (0.80-1.16) | 0.97 (0.81-1.18) | 0.104 |
| aHR (95% CI)^c^ | 1.00 (reference) | 1.16 (0.97-1.39) | 0.94 (0.78-1.14) | 0.94 (0.78-1.14) | 0.067 |
| Stroke |  |  |  |  |  |
| PY | 54,346 | 54,626 | 59,119 | 55,757 |  |
| Event (%) | 290 (3.6) | 300 (3.7) | 354 (4.0) | 339 (4.0) |  |
| Incidence/1,000 PY | 5.3 | 5.5 | 6.0 | 6.1 |  |
| HR (95% CI) | 1.00 (reference) | 1.03 (0.88-1.21) | 1.12 (0.96-1.31) | 1.14 (0.98-1.34) | 0.267 |
| aHR (95% CI)^a^ | 1.00 (reference) | 1.02 (0.87-1.20) | 1.10 (0.94-1.28) | 1.09 (0.93-1.28) | 0.565 |
| aHR (95% CI)^b^ | 1.00 (reference) | 1.03 (0.88-1.21) | 1.11 (0.95-1.30) | 1.11 (0.95-1.30) | 0.460 |
| aHR (95% CI)^c^ | 1.00 (reference) | 1.02 (0.87-1.20) | 1.10 (0.94-1.28) | 1.08 (0.92-1.26) | 0.643 |

HR calculated using the Cox proportional hazards model.

^a^Adjusted for age.

^b^Adjusted for age, household income, body mass index, hypertension, diabetes mellitus, dyslipidemia, smoking, alcohol consumption, moderate-to-vigorous physical activity, and Charlson comorbidity index.

^c^Adjusted for age, household income, body mass index, hypertension, diabetes mellitus, dyslipidemia, smoking, alcohol consumption, moderate-to-vigorous physical activity, Charlson comorbidity index, and total cholesterol.

Acronyms: PY, person-year; HR, hazard ratio; CI, confidence interval; aHR, adjusted hazard ratio.

**Supplemental Table 3.** Association of high-density lipoprotein cholesterol quartiles with incident cardiovascular disease among women

|  | **1^st^ quartile (n=11,865)** | **2^nd^ quartile (n=9,297)** | **3^rd^ quartile (n=12,172)** | **4^th^ quartile (n=10,386)** | ***P* for trend** |
| --- | --- | --- | --- | --- | --- |
| Range, mg/dL | ≤63 | 64-67 | 68-75 | ≥76 |  |
| Cardiovascular disease |  |  |  |  |  |
| PY | 80,325 | 63,037 | 82,438 | 70,562 |  |
| Event (%) | 580 (4.9) | 447 (4.8) | 578 (4.7) | 472 (4.5) |  |
| Incidence/1,000 PY | 7.2 | 7.1 | 7.0 | 6.7 |  |
| HR (95% CI) | 1.00 (reference) | 0.98 (0.87-1.11) | 0.97 (0.87-1.09) | 0.93 (0.82-1.05) | 0.657 |
| aHR (95% CI)^a^ | 1.00 (reference) | 1.01 (0.90-1.15) | 1.03 (0.92-1.16) | 1.01 (0.90-1.14) | 0.952 |
| aHR (95% CI)^b^ | 1.00 (reference) | 1.02 (0.90-1.15) | 1.06 (0.94-1.19) | 1.04 (0.92-1.18) | 0.807 |
| aHR (95% CI)^c^ | 1.00 (reference) | 1.02 (0.90-1.15) | 1.05 (0.93-1.18) | 1.02 (0.90-1.16) | 0.885 |
| Coronary heart disease |  |  |  |  |  |
| PY | 81,462 | 63,880 | 83,557 | 71,452 |  |
| Event (%) | 204 (1.7) | 158 (1.7) | 208 (1.7) | 147 (1.4) |  |
| Incidence/1,000 PY | 2.5 | 2.5 | 2.5 | 2.1 |  |
| HR (95% CI) | 1.00 (reference) | 0.99 (0.80-1.22) | 0.99 (0.82-1.21) | 0.82 (0.67-1.02) | 0.233 |
| aHR (95% CI)^a^ | 1.00 (reference) | 1.02 (0.83-1.25) | 1.05 (0.87-1.28) | 0.89 (0.72-1.10) | 0.471 |
| aHR (95% CI)^b^ | 1.00 (reference) | 1.03 (0.84-1.27) | 1.09 (0.90-1.32) | 0.94 (0.76-1.16) | 0.564 |
| aHR (95% CI)^c^ | 1.00 (reference) | 1.03 (0.83-1.26) | 1.07 (0.88-1.30) | 0.90 (0.73-1.12) | 0.453 |
| Stroke |  |  |  |  |  |
| PY | 80,950 | 63,484 | 83,025 | 70,893 |  |
| Event (%) | 396 (3.3) | 298 (3.2) | 393 (3.2) | 335 (3.2) |  |
| Incidence/1,000 PY | 4.9 | 4.7 | 4.7 | 4.7 |  |
| HR (95% CI) | 1.00 (reference) | 0.96 (0.83-1.12) | 0.97 (0.84-1.11) | 0.96 (0.83-1.12) | 0.941 |
| aHR (95% CI)^a^ | 1.00 (reference) | 0.99 (0.85-1.15) | 1.04 (0.90-1.19) | 1.06 (0.92-1.22) | 0.825 |
| aHR (95% CI)^b^ | 1.00 (reference) | 1.00 (0.86-1.16) | 1.05 (0.92-1.21) | 1.08 (0.93-1.25) | 0.665 |
| aHR (95% CI)^c^ | 1.00 (reference) | 0.99 (0.86-1.16) | 1.05 (0.91-1.21) | 1.07 (0.92-1.24) | 0.740 |

HR calculated using the Cox proportional hazards model.

^a^Adjusted for age.

^b^Adjusted for age, household income, body mass index, hypertension, diabetes mellitus, dyslipidemia, smoking, alcohol consumption, moderate-to-vigorous physical activity, and Charlson comorbidity index.

^c^Adjusted for age, household income, body mass index, hypertension, diabetes mellitus, dyslipidemia, smoking, alcohol consumption, moderate-to-vigorous physical activity, Charlson comorbidity index, and total cholesterol.

Acronyms: PY, person-year; HR, hazard ratio; CI, confidence interval; aHR, adjusted hazard ratio.

**Supplemental Table 4.** Association of change in high-density lipoprotein cholesterol levels with incident cardiovascular disease among men

|  | **1^st^ quartile (n=8,727)** | **2^nd^ quartile (n=7,619)** | **3^rd^ quartile (n=8,337)** | **4^th^ quartile (n=8,723)** | ***P* for trend** |
| --- | --- | --- | --- | --- | --- |
| Range, mg/dL | ≤-1 | 0 to +6 | +7 to +14 | ≥+15 |  |
| Cardiovascular disease |  |  |  |  |  |
| PY | 57,913 | 50,824 | 55,266 | 57,094 |  |
| Event (%) | 516 (5.9) | 444 (5.8) | 521 (6.2) | 636 (7.3) |  |
| Incidence/1,000 PY | 8.9 | 8.7 | 9.4 | 11.1 |  |
| HR (95% CI) | 1.00 (reference) | 0.98 (0.86-1.11) | 1.06 (0.94-1.20) | 1.25 (1.12-1.41) | <0.001 |
| aHR (95% CI)^a^ | 1.00 (reference) | 1.01 (0.89-1.14) | 1.07 (0.95-1.21) | 1.21 (1.08-1.36) | 0.004 |
| aHR (95% CI)^b^ | 1.00 (reference) | 1.02 (0.90-1.15) | 1.07 (0.95-1.21) | 1.17 (1.04-1.31) | 0.045 |
| aHR (95% CI)^c^ | 1.00 (reference) | 1.01 (0.89-1.15) | 1.06 (0.94-1.20) | 1.15 (1.02-1.29) | 0.080 |
| Coronary heart disease |  |  |  |  |  |
| PY | 58,827 | 51,461 | 56,041 | 58,048 |  |
| Event (%) | 204 (2.3) | 195 (2.6) | 232 (2.8) | 292 (3.3) |  |
| Incidence/1,000 PY | 3.5 | 3.8 | 4.1 | 5.0 |  |
| HR (95% CI) | 1.00 (reference) | 1.09 (0.90-1.33) | 1.19 (0.99-1.44) | 1.45 (1.21-1.74) | <0.001 |
| aHR (95% CI)^a^ | 1.00 (reference) | 1.11 (0.92-1.36) | 1.21 (1.00-1.46) | 1.42 (1.19-1.70) | 0.001 |
| aHR (95% CI)^b^ | 1.00 (reference) | 1.12 (0.92-1.36) | 1.20 (0.99-1.45) | 1.35 (1.13-1.62) | 0.010 |
| aHR (95% CI)^c^ | 1.00 (reference) | 1.12 (0.92-1.36) | 1.19 (0.99-1.44) | 1.33 (1.11-1.60) | 0.015 |
| Stroke |  |  |  |  |  |
| PY | 58,480 | 51,392 | 55,992 | 57,930 |  |
| Event (%) | 332 (3.8) | 273 (3.6) | 311 (3.7) | 367 (4.2) |  |
| Incidence/1,000 PY | 5.7 | 5.3 | 5.6 | 6.3 |  |
| HR (95% CI) | 1.00 (reference) | 0.94 (0.80-1.10) | 0.98 (0.84-1.14) | 1.12 (0.96-1.30) | 0.128 |
| aHR (95% CI)^a^ | 1.00 (reference) | 0.97 (0.82-1.13) | 1.00 (0.85-1.16) | 1.07 (0.92-1.24) | 0.615 |
| aHR (95% CI)^b^ | 1.00 (reference) | 0.98 (0.84-1.15) | 1.00 (0.85-1.16) | 1.04 (0.90-1.21) | 0.890 |
| aHR (95% CI)^c^ | 1.00 (reference) | 0.98 (0.83-1.15) | 0.99 (0.85-1.16) | 1.03 (0.89-1.19) | 0.937 |

HR calculated using the Cox proportional hazards model.

^a^Adjusted for age.

^b^Adjusted for age, household income, body mass index, hypertension, diabetes mellitus, dyslipidemia, smoking, alcohol consumption, moderate-to-vigorous physical activity, and Charlson comorbidity index.

^c^Adjusted for age, household income, body mass index, hypertension, diabetes mellitus, dyslipidemia, smoking, alcohol consumption, moderate-to-vigorous physical activity, Charlson comorbidity index, and total cholesterol.

Acronyms: PY, person-year; HR, hazard ratio; CI, confidence interval; aHR, adjusted hazard ratio.

**Supplemental Table 5.** Association of change in high-density lipoprotein cholesterol levels with incident cardiovascular disease among women

|  | **1^st^ quartile (n=10,445)** | **2^nd^ quartile (n=10,936)** | **3^rd^ quartile (n=10,999)** | **4^th^ quartile (n=11,329)** | ***P* for trend** |
| --- | --- | --- | --- | --- | --- |
| Range, mg/dL | ≤-3 | -2 to +5 | +6 to +13 | ≥+14 |  |
| Cardiovascular disease |  |  |  |  |  |
| PY | 71,044 | 74,289 | 74,671 | 76,286 |  |
| Event (%) | 438 (4.2) | 483 (4.4) | 520 (4.7) | 635 (5.6) |  |
| Incidence/1,000 PY | 6.2 | 6.5 | 7.0 | 8.3 |  |
| HR (95% CI) | 1.00 (reference) | 1.06 (0.93-1.20) | 1.13 (1.00-1.28) | 1.35 (1.20-1.53) | <0.001 |
| aHR (95% CI)^a^ | 1.00 (reference) | 1.03 (0.91-1.18) | 1.09 (0.96-1.24) | 1.18 (1.04-1.33) | 0.039 |
| aHR (95% CI)^b^ | 1.00 (reference) | 1.03 (0.91-1.18) | 1.08 (0.95-1.22) | 1.15 (1.01-1.30) | 0.136 |
| aHR (95% CI)^c^ | 1.00 (reference) | 1.03 (0.91-1.18) | 1.07 (0.95-1.22) | 1.14 (1.01-1.28) | 0.187 |
| Coronary heart disease |  |  |  |  |  |
| PY | 71,855 | 75,262 | 75,649 | 77,508 |  |
| Event (%) | 149 (1.4) | 173 (1.6) | 176 (1.6) | 219 (1.9) |  |
| Incidence/1,000 PY | 2.1 | 2.3 | 2.3 | 2.8 |  |
| HR (95% CI) | 1.00 (reference) | 1.11 (0.89-1.38) | 1.12 (0.90-1.40) | 1.36 (1.11-1.68) | 0.023 |
| aHR (95% CI)^a^ | 1.00 (reference) | 1.09 (0.88-1.36) | 1.08 (0.87-1.35) | 1.20 (0.97-1.48) | 0.390 |
| aHR (95% CI)^b^ | 1.00 (reference) | 1.08 (0.87-1.35) | 1.06 (0.85-1.32) | 1.15 (0.93-1.41) | 0.642 |
| aHR (95% CI)^c^ | 1.00 (reference) | 1.08 (0.87-1.35) | 1.05 (0.85-1.31) | 1.12 (0.91-1.39) | 0.737 |
| Stroke |  |  |  |  |  |
| PY | 71,499 | 74,827 | 75,186 | 76,856 |  |
| Event (%) | 295 (2.8) | 326 (3.0) | 360 (3.3) | 440 (3.9) |  |
| Incidence/1,000 PY | 4.1 | 4.4 | 4.8 | 5.7 |  |
| HR (95% CI) | 1.00 (reference) | 1.06 (0.90-1.24) | 1.16 (1.00-1.35) | 1.39 (1.20-1.61) | <0.001 |
| aHR (95% CI)^a^ | 1.00 (reference) | 1.03 (0.88-1.21) | 1.12 (0.96-1.31) | 1.20 (1.04-1.40) | 0.057 |
| aHR (95% CI)^b^ | 1.00 (reference) | 1.04 (0.88-1.21) | 1.11 (0.95-1.29) | 1.18 (1.02-1.37) | 0.126 |
| aHR (95% CI)^c^ | 1.00 (reference) | 1.04 (0.88-1.21) | 1.11 (0.95-1.29) | 1.17 (1.01-1.36) | 0.147 |

HR calculated using the Cox proportional hazards model.

^a^Adjusted for age.

^b^Adjusted for age, household income, body mass index, hypertension, diabetes mellitus, dyslipidemia, smoking, alcohol consumption, moderate-to-vigorous physical activity, and Charlson comorbidity index.

^c^Adjusted for age, household income, body mass index, hypertension, diabetes mellitus, dyslipidemia, smoking, alcohol consumption, moderate-to-vigorous physical activity, Charlson comorbidity index, and total cholesterol.

Acronyms: PY, person-year; HR, hazard ratio; CI, confidence interval; aHR, adjusted hazard ratio.

**Supplemental Table 6.** Association of change in high-density lipoprotein cholesterol levels between 2009-2010 and 2013-2014 with incident cardiovascular disease

|  | **1^st^ quartile (n=6,140)** | **2^nd^ quartile (n=5,628)** | **3^rd^ quartile (n=6,227)** | **4^th^ quartile (n=6,109)** | ***P* for trend** |
| --- | --- | --- | --- | --- | --- |
| Range, mg/dL | ≤-1 | 0 to +7 | +8 to +16 | ≥+17 |  |
| Cardiovascular disease |  |  |  |  |  |
| HR (95% CI) | 1.00 (reference) | 1.01 (0.82-1.25) | 1.08 (0.88-1.31) | 1.38 (1.14-1.67) | 0.002 |
| aHR (95% CI)^a^ | 1.00 (reference) | 1.01 (0.82-1.24) | 1.05 (0.86-1.28) | 1.28 (1.06-1.55) | 0.028 |
| aHR (95% CI)^b^ | 1.00 (reference) | 1.00 (0.82-1.24) | 1.04 (0.85-1.27) | 1.24 (1.02-1.50) | 0.073 |
| aHR (95% CI)^c^ | 1.00 (reference) | 1.01 (0.82-1.24) | 1.04 (0.85-1.27) | 1.24 (1.03-1.50) | 0.071 |
| Coronary heart disease |  |  |  |  |  |
| HR (95% CI) | 1.00 (reference) | 0.85 (0.59-1.23) | 1.03 (0.73-1.45) | 1.59 (1.17-2.17) | <0.001 |
| aHR (95% CI)^a^ | 1.00 (reference) | 0.85 (0.59-1.22) | 1.00 (0.72-1.41) | 1.49 (1.09-2.03) | 0.003 |
| aHR (95% CI)^b^ | 1.00 (reference) | 0.84 (0.58-1.21) | 0.98 (0.70-1.38) | 1.41 (1.03-1.93) | 0.010 |
| aHR (95% CI)^c^ | 1.00 (reference) | 0.85 (0.59-1.22) | 0.99 (0.70-1.39) | 1.42 (1.04-1.94) | 0.010 |
| aHR (95% CI)^d^ | 1.00 (reference) | 1.10 (0.85-1.41) | 1.09 (0.85-1.39) | 1.20 (0.95-1.53) | 0.502 |
| Stroke |  |  |  |  |  |
| HR (95% CI) | 1.00 (reference) | 1.10 (0.86-1.41) | 1.13 (0.89-1.44) | 1.36 (1.08-1.72) | 0.061 |
| aHR (95% CI)^a^ | 1.00 (reference) | 1.10 (0.85-1.41) | 1.10 (0.87-1.41) | 1.26 (0.99-1.59) | 0.280 |
| aHR (95% CI)^b^ | 1.00 (reference) | 1.10 (0.85-1.41) | 1.10 (0.86-1.40) | 1.23 (0.97-1.55) | 0.393 |
| aHR (95% CI)^c^ | 1.00 (reference) | 1.10 (0.86-1.41) | 1.10 (0.87-1.40) | 1.23 (0.97-1.55) | 0.386 |
| aHR (95% CI)^d^ | 1.00 (reference) | 0.81 (0.56-1.18) | 0.91 (0.65-1.29) | 1.28 (0.93-1.77) | 0.043 |

HR calculated using the Cox proportional hazards model.

^a^Adjusted for age and sex.

^b^Adjusted for age, sex household income, body mass index, hypertension, diabetes mellitus, dyslipidemia, smoking, alcohol consumption, moderate-to-vigorous physical activity, and Charlson comorbidity index.

^c^Adjusted for age, sex, household income, body mass index, hypertension, diabetes mellitus, dyslipidemia, smoking, alcohol consumption, moderate-to-vigorous physical activity, Charlson comorbidity index, and total cholesterol.

^d^Assessed using the competing risk model to calculate the subdistribution hazard ratio after adjustments for variables in the model C with coronary heart disease or stroke as competing risks for each other.

Acronyms: PY, person-year; HR, hazard ratio; CI, confidence interval; aHR, adjusted hazard ratio.

**Supplemental Table 7.** Sensitivity analyses on association of change in high-density lipoprotein cholesterol levels with incident cardiovascular disease

|  | **1^st^ quartile** | **2^nd^ quartile** | **3^rd^ quartile** | **4^th^ quartile** | ***P* for trend** |
| --- | --- | --- | --- | --- | --- |
| 1-year | 1.00 (reference) | 1.04 (0.95-1.15) | 1.07 (0.98-1.18) | 1.12 (1.02-1.23) | 0.096 |
| 2-year | 1.00 (reference) | 1.03 (0.93-1.15) | 1.08 (0.97-1.19) | 1.13 (1.02-1.24) | 0.105 |
| 3-year | 1.00 (reference) | 1.04 (0.93-1.16) | 1.10 (0.99-1.24) | 1.15 (1.03-1.28) | 0.058 |

HR calculated using the Cox proportional hazards model after adjustments for age, sex, household income, body mass index, hypertension, diabetes mellitus, dyslipidemia, smoking, alcohol consumption, moderate-to-vigorous physical activity, Charlson comorbidity index, and total cholesterol.

**Supplemental Table 8.** Association of change in high-density lipoprotein cholesterol levels between 2009-2010 and 2013-2014 with incident cardiovascular disease according to the change in low-density lipoprotein cholesterol levels between 2009-2010 and 2013-2014

|  | **Change in HDL-C (mg/dL)** | | | | ***P* for trend** |
| --- | --- | --- | --- | --- | --- |
|  | **1^st^ quartile,**  **≤-1** | **2^nd^ quartile,**  **0 to +7** | **3^rd^ quartile,**  **+8 to +16** | **4^th^ quartile,**  **≥+17** |  |
| Cardiovascular disease | |  |  |  |  |
| LDL-C decrease^a^ |  |  |  |  |  |
| aHR (95% CI) | 1.00 (reference) | 0.89 (0.66-1.22) | 1.01 (0.76-1.34) | 1.14 (0.87-1.50) | 0.391 |
| *P* value |  | 0.482 | 0.969 | 0.345 |  |
| LDL-C increase^b^ |  |  |  |  |  |
| aHR (95% CI) | 1.00 (reference) | 1.12 (0.85-1.48) | 1.07 (0.81-1.41) | 1.39 (1.06-1.82) | 0.081 |
| *P* value |  | 0.435 | 0.653 | 0.016 |  |
| Coronary heart disease | |  |  |  |  |
| LDL-C decrease^c^ |  |  |  |  |  |
| aHR (95% CI) | 1.00 (reference) | 0.89 (0.61-1.31) | 1.03 (0.72-1.46) | 1.05 (0.75-1.48) | 0.831 |
| *P* value |  | 0.562 | 0.881 | 0.777 |  |
| LDL-C increase^d^ |  |  |  |  |  |
| aHR (95% CI) | 1.00 (reference) | 1.29 (0.92-1.81) | 1.14 (0.81-1.60) | 1.42 (1.02-1.99) | 0.185 |
| *P* value |  | 0.135 | 0.467 | 0.039 |  |
| Stroke |  |  |  |  |  |
| LDL-C decrease^c^ |  |  |  |  |  |
| aHR (95% CI) | 1.00 (reference) | 0.83 (0.48-1.42) | 0.93 (0.57-1.54) | 1.26 (0.80-1.99) | 0.306 |
| *P* value |  | 0.495 | 0.787 | 0.317 |  |
| LDL-C increase^d^ |  |  |  |  |  |
| aHR (95% CI) | 1.00 (reference) | 0.80 (0.48-1.34) | 0.88 (0.54-1.44) | 1.34 (0.85-2.10) | 0.174 |
| *P* value |  | 0.400 | 0.612 | 0.207 |  |

Data are hazard ratio (95% confidence interval) calculated using the Cox proportional hazards model after adjustments for age, sex, household income, body mass index, hypertension, diabetes mellitus, dyslipidemia, smoking, alcohol consumption, moderate-to-vigorous physical activity, and Charlson comorbidity index.

^a^With change in LDL-C level of <0 in the second health screening (2013-2014) compared to the first health screening (2009-2010).

^b^With change in LDL-C level of ≥0 in the second health screening (2013-2014) compared to the first health screening (2009-2010).

^c^Competing risk analysis for the model A with coronary heart disease or stroke as competing risks for each other.

^d^Competing risk analysis for the model B with coronary heart disease or stroke as competing risks for each other.

Acronyms: HDL-C, high-density lipoprotein cholesterol; LDL-C, low-density lipoprotein cholesterol.
